# Oxygen Vacancies Can Drive Surface Transformation of High-Entropy Perovskite Oxide for the Oxygen Evolution Reaction as Probed with Scanning Probe Microscopy

**DOI:** 10.1021/acsami.4c22352

**Published:** 2025-04-03

**Authors:** Michael Verhage, Stijn J.H.M. van den Broek, Christ Weijtens, Cornelis F. J. Flipse

**Affiliations:** Molecular Materials and Nanosystems (M2N), Department of Applied Physics and Science Education, Eindhoven University of Technology, Eindhoven 5600 MB, the Netherlands

**Keywords:** high entropy perovskite oxide, scanning tunneling microscopy, thin film, surface transformation, oxygen evolution reaction.

## Abstract

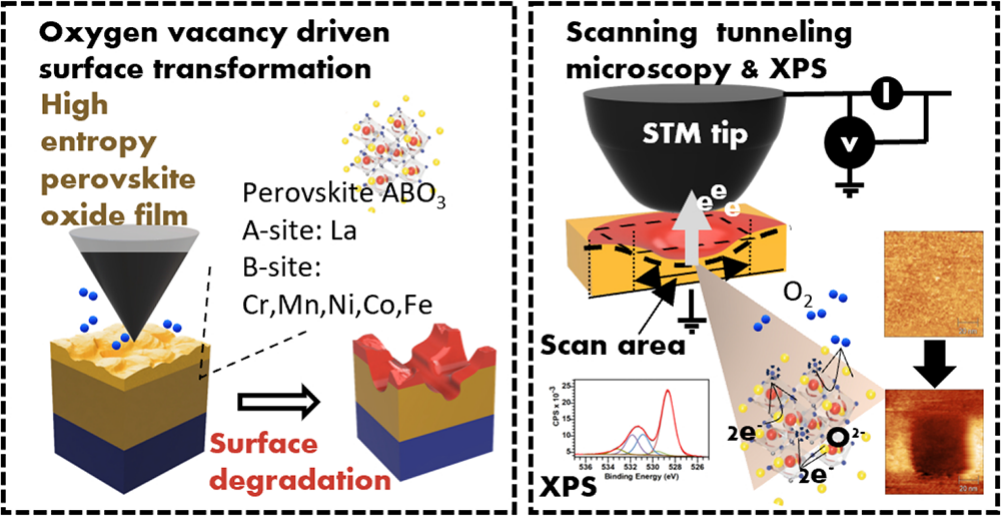

Epitaxial transition-metal oxide perovskite catalysts form a highly active catalyst class for the oxygen evolution reaction (OER). Understanding the origin of chemical dissolution and surface transformations during the OER is important to rationally design effective catalyst. These changes arise from complex interactions involving dynamic restructuring and electronic/structural adaptations. Although initial instability is common, surfaces can reach equilibrium through chemical transformations. High entropy perovskite oxides (HEPOs), which incorporate multiple 3d metal cations in near-equimolar ratios, have emerged as promising catalysts due to their enhanced OER activity compared to single-cation variants, attributed to their high configurational entropy and compositional flexibility. To advance HEPO catalyst applications, understanding the mechanisms governing their surface (in)stability is important. In this work, we examine surface degradation in epitaxial La(Cr,Mn,Fe,Co,Ni)O_3–*δ*_ thin films before and after OER using complementary scanning tunneling microscopy (STM) and X-ray photoelectron spectroscopy (XPS). STM reveals tip-induced degradation of as-grown films under positive bias, attributed to oxygen anion removal and charge trapping-induced lattice degradation, demonstrating its utility as a probe for surface stability dynamics. Post-OER XPS analysis shows irreversible surface transformations from the initial epitaxial phase, characterized by 3d-metal leaching and formation of La and d-metal (oxy)hydroxides. Our findings indicate that oxygen vacancies and lattice strain trigger structural breakdown in these multi-cation perovskite surfaces during the OER, leading to surface restructuring and diminished catalytic performance compared to the as-grown epitaxial HEPO phase. This work identifies oxygen leaching as the likely primary driver of surface transformation during the OER. We show that STM offers an important tool to probe the transformation even before operando conditions, which can find use in similar material studies.

## Introduction

High entropy oxides (HEOs) represent a novel category of materials characterized by a range of unique properties and applications, such as electrochemistry, memresistors and thermoelectrics. These materials are stabilized by high configurational entropy which maintains their structure through a multitude of elemental combinations and arrangements. In addition to rare earth based HEOs, numerous other compositions have been developed in recent years, including HEO fluorides,^1^ alkaline earth metals,^2^ and halides.^3^ These HEOs attract interest for various applications such as electrochemistry^4,5^ and batteries.^6^

HEO’s have also shown remarkable effectiveness as electrocatalysts for the oxygen evolution reaction (OER).^4,5,7,8^ Recently, Kante et al.^9^ have demonstrated an efficient OER catalyst in the form of an epitaxial thin-film high entropy perovskite oxide La(Cr,Mn,Fe,Co,Ni)O_3-δ_ (HEPO), which showed a substantial initial increase in activity compared to the conventional LaNiO_3_ (LNO) epitaxial thin-film catalyst. This notable improvement in activity calls for further research into its stability, as the relationship between stability and activity often suggests a compromise between the two.^10^

The large number of elements in HEPO is considered to make the reaction more favorable by reducing the reaction barrier, promoting synergy between metal sites,^9^ and enhancing the overlap between the oxygen 2p and metal 3d orbitals^11^ and *e_g_* broadening,^12^ leading to more efficient charge transfer and proton removal of the first step in the OER cycle.^13^ However, during OER, the surfaces of metal oxide perovskite oxides tends to degrade and reconstruct.^14,15^ The leaching of for example oxygen during the reaction can result in surface degradation, frequently accompanied by a decline in activity.^16^ The high configuration entropy of a HEPO and the variation in B-site ionic radii leads to severe crystal lattice distortion which benefits oxygen vacancy creation and formation by strain relaxation.^17,18^ New compounds can form from these elements during the reaction. In a way of mimicking survival bias, only compounds that do not dissolve in an alkaline electrolyte are retained on the surface, not necessarily in crystalline form. Hence, the beneficial properties of the epitaxial thin film HEPO may be lost if the surface transforms.

We studied an epitaxial thin film of a HEPO with the composition of La(Cr,Mn,Fe,Co,Ni)O_3–δ_, similar to that of the work of Kante et al.,^9^ with a thickness of 10 nm which was deposited on Nb:SrTiO_3_ (NSTO) by means of pulsed laser deposition (PLD).^9^ In this work, we examine surface modifications of this HEPO pre- and post-OER cyclic voltammetry (CV) using both scanning probe microscopy (SPM) and X-ray photoemission spectroscopy (XPS). We show with SPM that the surface undergoes noticeable changes following OER. Its morphology changes from a flat, but corrugated structure to a rough surface. This morphological change coincides with a chemical transformation into a La- and 3d-metal(oxy)hydroxide-rich surface. This metal(oxy)hydroxide has also been reported to form on the surface of nickelates.^19^ Furthermore, of the 3d elements in the HEPO film, only Ni and Co persist in significant amounts, both forming likely (oxy)hydroxides. The other elements, Fe, Mn, and Cr, have been depleted from the surface, probably migrating into the electrolyte during OER cycling.

Using scanning tunneling microscopy (STM), we demonstrate that a positive tip bias can lead to the surface decomposition and degradation, which is conjectured to be driven by oxygen anion diffusion under the strong local electric field (10^9^ V m^–1^).^20^ The resulting accumulation of oxygen vacancies on the surface, acts as traps for charge. The trapped charge likely further distort the oxygen octahedra, leading to an eventual collapse of the crystal. Such surface transformation may indicate that similar oxygen leaching occurs at the surface during OER. STM provides distinct evidence pointing to the strong local electric field as the driver for oxygen diffusion to the surface. Oxygen mobility and diffusion^21^ in oxides have been reported to be possible at room temperature and correlated with topotactic transformation.^22−24^ Oxygen vacancies can cause the lattice to expand and impact its elasticity.^25^ The capture of charge at these defects may ultimately result in the crystal’s complete breakdown, and can potentially form polarons which also deform the lattice structure.^26,27^ These observations could help better understand why many metal oxide perovskites degrade during OER, especially at high current densities.

STM is a valuable method for probing the early stages of surface changes in perovskite epitaxial thin film catalysts even before they are subjected to OER conditions. Future investigations might leverage STM as a valuable technique to evaluate the surface stability and transformation potential of these catalysts. STM also offers insights into the effects of local charge trapping on crystal structures and degradation. Furthermore, due to its ability to generate intense local electric fields, STM could potentially help study the influence of electric fields, such as those in the double layer,^28^ on surface stability. The application of STM in liquid environments^29^ would further boast its applicability for operando conditions studies of surface transformations.

## Results and Discussion

### Film Characterization

**Figure 1:**
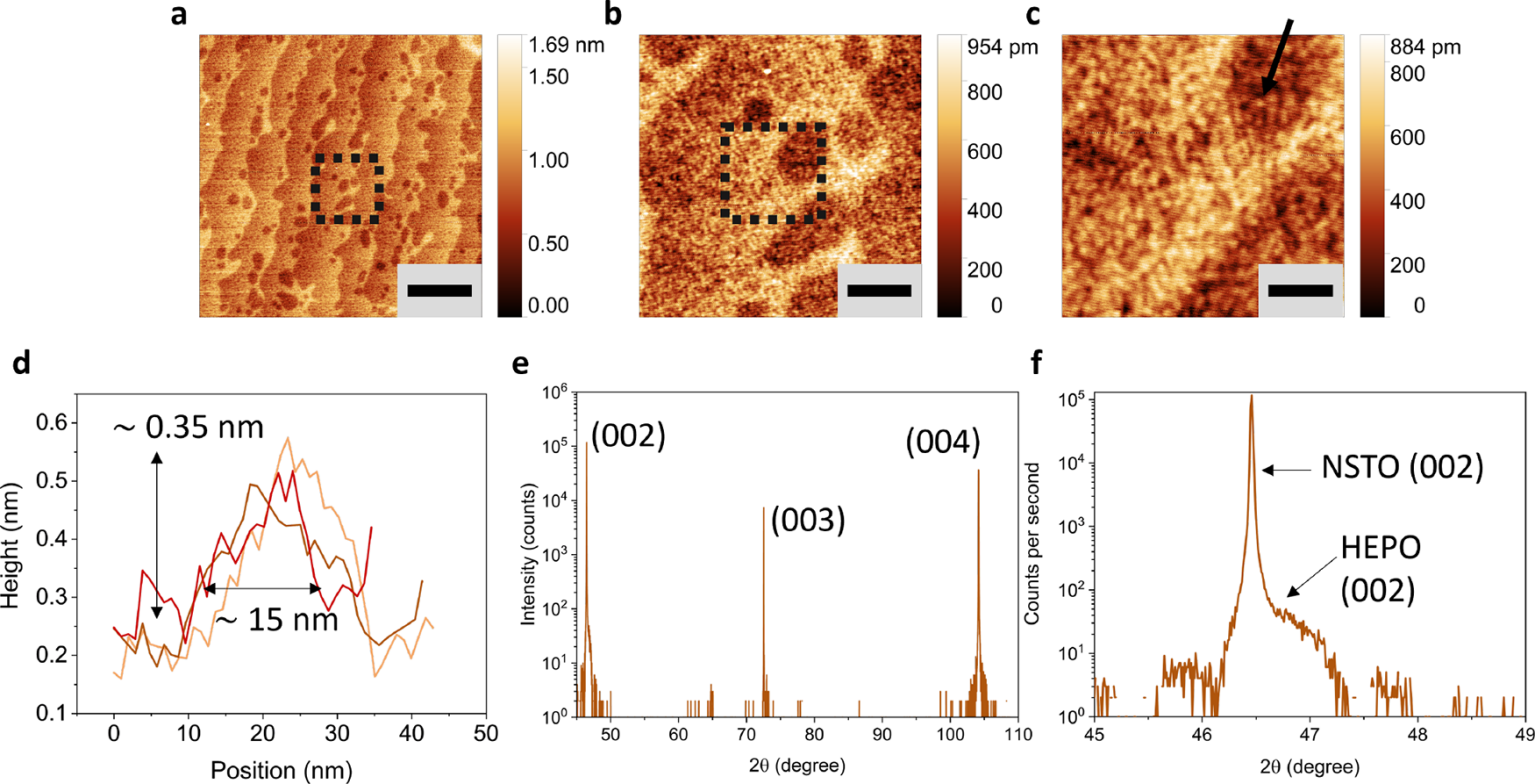
HEPO characterization pre-OER. (a) Large scale AFM image showing stepped surface with a flat surface. The holes near the step-edge originate from clearing procedure of the NSTO substrate and were noted before film deposition on similar substrates. The scale bar is equal to 1 μm. (b, c) High resolution AFM images, taken at the areas of (a, b) indicated by the black dotted box, showing the circular surface features of the film. The scale bar is equal to 200 and 50 nm, respectively. (d) Height profile of surface features, taken at a location indicated by the black arrow in (c), showing single unit cell height over several nanometer wide structures, indicating epitaxial growth. (e) XRD 0D detector wide-range scan (2θ) showing predominant NSTO peaks. (f) High-resolution XRD peak (2θ) showing a shoulder right to the NSTO (002) peak, indicating epitaxial growth of the HEPO film in the perovskite lattice orientation direction (001).

In our earlier studies, we observed that PLD-grown thin films, such as Manganite LaSrMnO_3_^30^ and nickelate LaNiO_3_,^31^ exhibit nanoscale corrugations that are often missed when using lower-resolution AFM with micrometer-scale imaging. Epitaxial metal oxide perovskite films are often considered atomically flat. According to our criteria, films must exhibit a roughness less than the height of one atomic layer (2 Å) to be considered atomically flat. In Figure 1a–c, AFM images captured at increasing magnifications reveal a relative flat surface morphology, but not atomically smooth. The observed plateaus on the film are attributed to the vicinally cut NSTO substrate, while the holes near the step-edges result from the NSTO substrate cleaning process preceding PLD deposition.^9^ High-resolution AFM imaging in Figure 1c demonstrates that the surface exhibits nanoscale corrugations. A feature, indicated by the black arrow, shows that these corrugations are composed of circular-like structures with an average height of 0.35 nm; close to the height of one unit cell, Figure 1d. The width of these formations spans several nanometers, implying that they are probably single-layer flat regions formed during the layer-by-layer growth of the film. We have documented similar growth patterns for the Manganite,^30^ driven by the kinetic movement of adatoms on the surface that encounter energetic barriers, particularly near the vicinal steps.

The film quality as characterized by XRD is similar to the work of Kante et al.,^9^ with a similar thickness of 10 nm. We employed XRD using a 0D detector scan mode (2θ) in Figure 1e,f, shows the epitaxial nature of the film with a shoulder (002) peak besides the NSTO (002) peak. The HEPO film peak partially overlap with the NSTO substrate peaks, as given in Figure 1f, indicating epitaxial growth in the (001) orientation of the perovskite lattice structure. The XPS characterization of the film will be discussed later. For further XRD details about the film we refer to the work of Kante et al.^9^

### Investigation of HEPO Surface Degradation Utilizing STM

**Figure 2:**
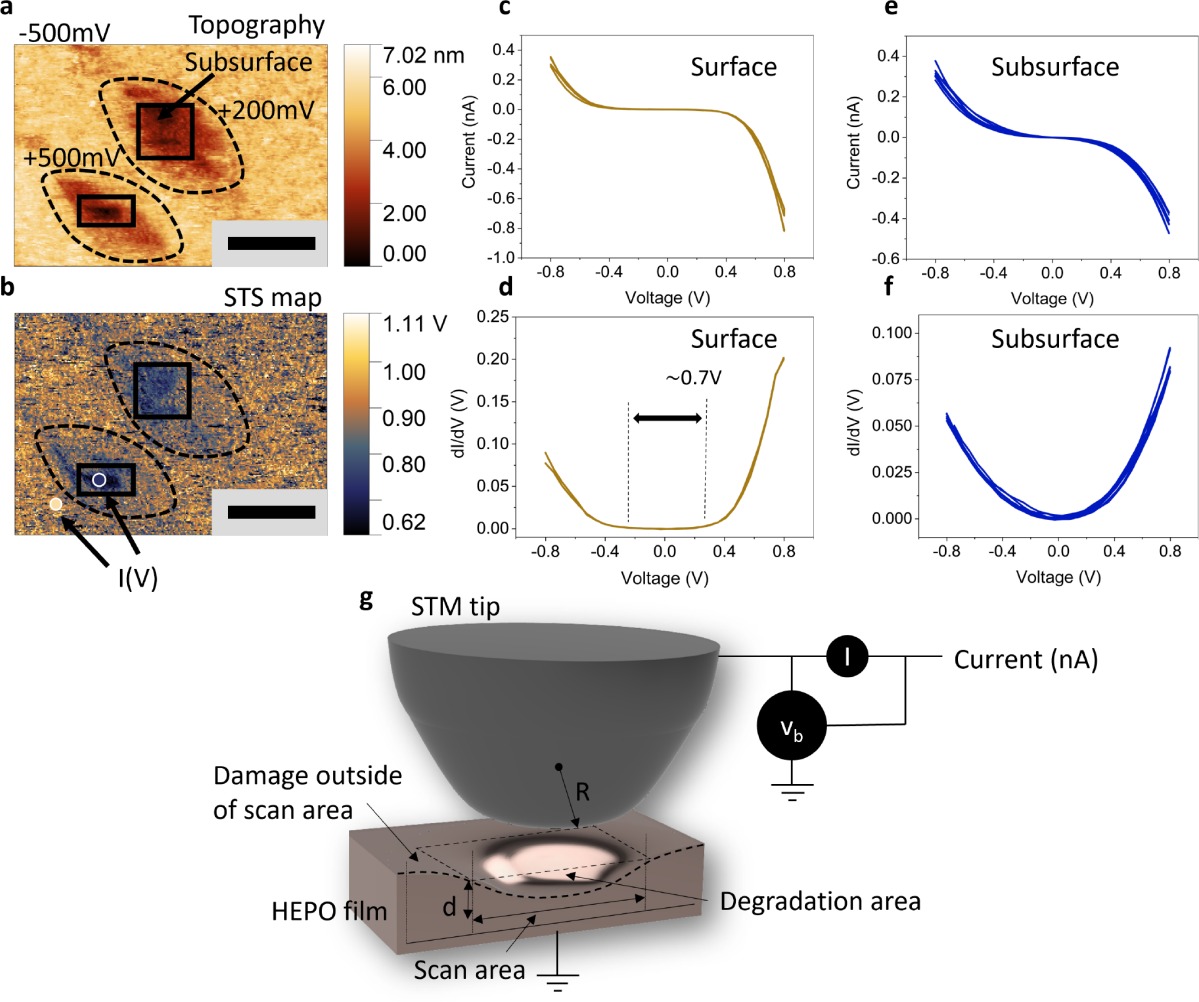
Surface degradation probed with STM. (a) An STM topography image sized 400 × 300 nm, acquired using a gap voltage of −500 mV and a current set point of 300 pA. The image reveals two areas, marked by black lines, previously scanned at +200 and +500 mV. A depression in surface height *d* is observed locally, which we infer as degradation exposing the film’s subsurface. The degradation area extends beyond the square regions marked by the dashed black line. (b) Correlated STS mapping of (a). The as-grown surface, shown in yellow, exhibits an increased (at +500 mV) LDOS compared to the blue-colored degraded subsurface, indicating a p-type semiconducting surface. Two regions are marked by dots for *I*(*V*) and d*I*/d*V* spectroscopy. (c–f) *I*(*V*) and d*I*/d*V* curves indicating the disappearance of the 0.7 V electronic gap, suggesting an alteration in electronic properties due to STM scanning with positive bias voltage. (g) A schematic illustration of surface degradation extending beyond the scan area. With the sample grounded and tip biased, a positive bias (*V*_*b*_) facilitates the extraction of oxygen anions and the leaching of oxygen into the vacuum chamber. Because of the finite tip radius *R*, the damage extends beyond the scanned area indicating contribution of the electric field (gradient) to the degradation process. The strength of degradation depth *d* is reduced outside of the scan area. The main influence of surface degradation is by the tunneling current *I*.

Using STM, the surface of the HEPO thin film can be imaged and analyzed for factors that influence stability and degradation prior to OER. We project these properties on the OER operation characteristics, especially in light of surface transformations. For example, the application of a local pulsed voltage between the STM tip and the surface has proven effective in altering the surface’s conductive state of perovskite thin films driven by the creation, annihilation, and migration of oxygen vacancies^32^ at room temperature (RT). In previous research,^31^ we showed that the epitaxial thin film LaNiO_3_ surface can deteriorate under the influence of small bias voltages at RT. This degradation arises from the formation and diffusion of oxygen vacancies under the influence of the electric field (gradient) and breaking of the Ni–O bond with charge trapping in the confined Ni 3d states. The behavior is particularly notable in ultrahigh vacuum conditions, where the oxygen loss cannot be reversed, making surface degradation rapidly measurable. For our epitaxial HEPO film, a similar tip-induced surface degradation was observed. In Figure 2a, the surface shows locally induced damage in two scanned areas. These areas were scanned at tip voltages of +200 mV and +500 mV, marked by black square boxes in the figure. In particular, significant damage is evident within these scanned areas by creating a square depression on the surface, corresponding to the scanned area with the tip. Applying a negative tip voltage does not incur significant surface damage. We note that the degradation-affected area can exceed the scanned regions, as shown by the black dotted lines, suggesting that the tunneling current magnitude between the STM tip and the surface is not the only sole cause of the damage, as the tip did not image beyond the square areas. Furthermore, the tip has a radius *R* on the order of 200 nm, which is larger than the scanned area. In Supplementary S1, a similar experiment using a sharper tip, i.e. smaller radius *R*, demonstrates better imaging resolution and a more confined degradation area, indicating a more localized electric field gradient effect as expected from a sharp tip.

The tip-induced deterioration of the film’s surface is likely to modify its structure and thus affecting its electronic properties. In the case of LNO we showed^31^ that the local density of states (LDOS) can transition from metallic to semiconducting by tip-induced degradation, a change hypothesized to arise from the creation of oxygen vacancies, as validated by photoemission spectroscopy experiments by other authors^33^ and DFT calculations.^34^ We evaluated the effect of this degradation on the LDOS of the HEPO thin film by simultaneously mapping with scanning tunneling spectroscopy (STS) in Figure 2b. The subsurface, revealed by tip-induced degradation, exhibits a change in LDOS relative to the unperturbed surface (yellow). Increased electrical conductivity is extremely important in electrocatalytic OER activity of the perovskite oxide thin films.^35^ For HEOs^36^ and LaFeO_3–*δ*_^37^ for example, the increased OER activity has been related to an increase in conductivity by oxygen vacancies donating electrons to the conduction band.^38^ In Figure 2b we observe a topographic correlation with the tip-induced damage of Figure 2a, as accompanied by a local change in LDOS compared to the unperturbed surface. Furthermore, with STS as a single location within the damaged area (blue point) and outside the damaged area (yellow point), we observe a stark LDOS difference, Figure 2c–f. The STS data demonstrate that the initial surface has an electronic gap of approximately 0.7 eV, exhibiting a higher LDOS on the positive voltage side. This semiconducting property suggests an p-type character of the HEPO film surface. However, the surface also has some hydroxylation from exposure to ambient conditions prior to the STM experiments, as also supported by XPS later on. Conversely, the subsurface regions affected by the tip’s positive bias exhibit a semimetallic state and the disappearance of the electronic gap of the insulating bulk. We attribute this transformation of the LDOS to the creation of a significant amount of oxygen vacancies, which can introduce free electrons into the conduction band^38^ at high concentration (for a p-type oxide) by reducing the hole concentration, in contrast to negative charge transfer nickelates,^39^ which become more insulating by increasing the concentration of oxygen vacancies.^40^ The schematic in Figure 2g illustrates the STM tip-induced degradation area. The area of degradation is larger than the tip radius *R*.

**Figure 3:**
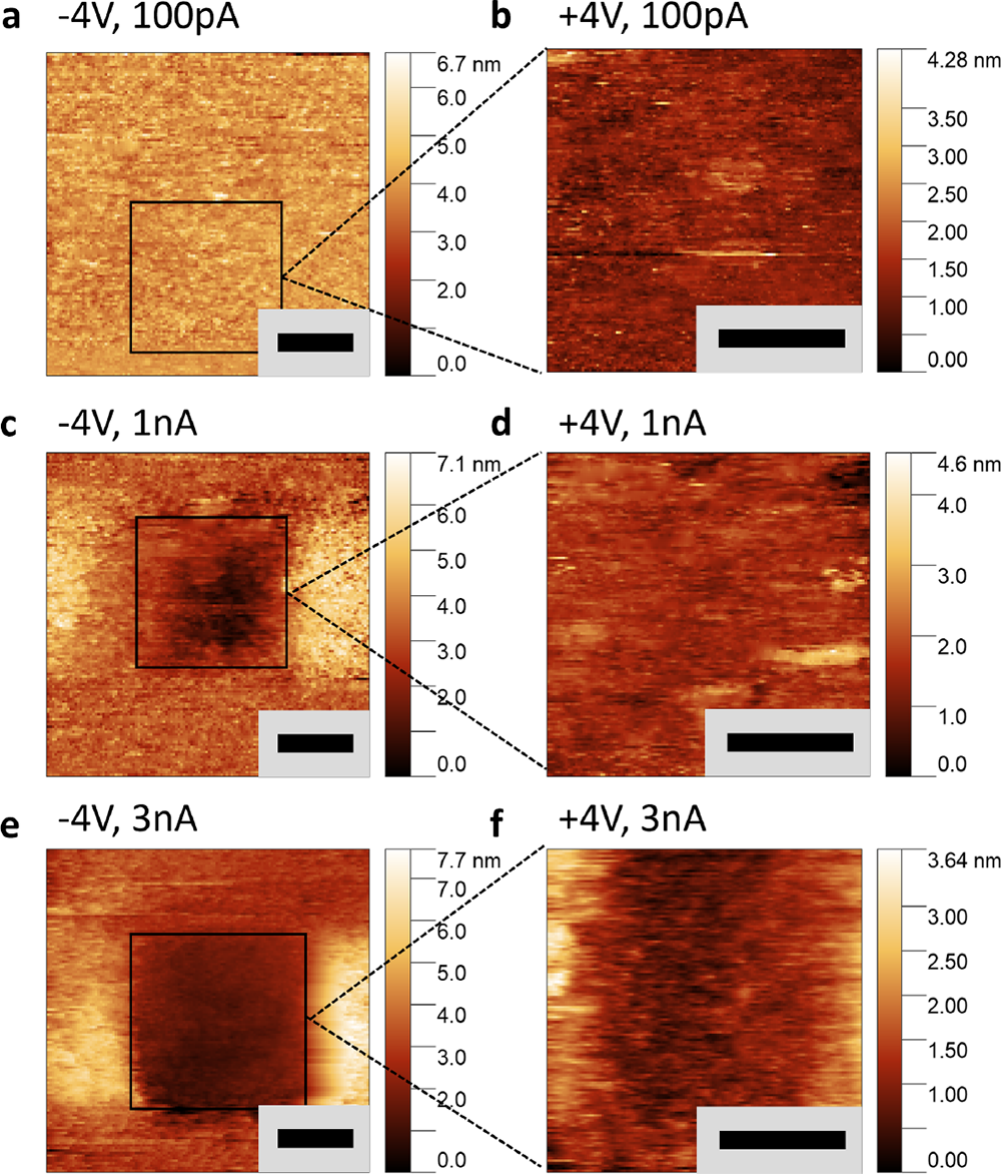
Tip-induced degradation as a function of tunnel current magnitude. (a, c, e) Large scale 100 × 100 nm topographic map imaged with a negative gap voltage of −4 V with varying tunneling current set point magnitudes. The images were taken after local scanning with positive bias voltages (b, d, f) with varying tunneling current set point magnitudes, and positive bias voltage. Local degradation is imaged, where care was taken to minimize out-side scan window damage using a sharp tip. At increased current magnitude set points, i.e., 3 nA, the degradation is strongest. Negative bias does not incur tip-induced bias, while positive bias does. Combined with the current magnitude, the degradation effect can be controlled with the STM tip. In all images the black scale bar is equal to 20 nm.

The magnitude of the tunneling current was identified as the key factor influencing the strength of surface degradation caused by the STM tip. In Figure 3, the tunneling current set points were varied between 100 pA and 3 nA, while maintaining a high bias voltage of ± 4 V during scanning. This high bias provided a substantial LDOS contribution for tunneling, see Figure 2c compared to the smaller bias voltages used earlier, resulting in increased tip–sample distances (i.e., >1 nm, that mitigate a potential physical force contributing to tip-induced damage. When the current set point is set to 100 pA, there is no significant surface degradation within a single scan area, as shown in Figure 3a,b. However, a significant increase in the degradation effect associated with higher currents was imaged. At 1 nA Figure 3c,d, noticeable area of degradation sized with the scan window were observed, which became more pronounced at 3 nA, Figure 3e,f. Since the areas were scanned with a less perturbative negative bias at the same current density to examine tip-induced damage, we can rule out Joule heating as a cause of degradation.

The degree of surface degradation is also influenced by the magnitude of the tip’s bias voltage and the local electric field (gradient).^31^Supplementary S2 illustrates the correlation between the bias voltage and surface deterioration. Several 50 × 50 nm scans are performed with gap voltages ranging from +300 to +700 mV, followed by imaging with a larger 100 × 100 nm scan at −700 mV. The depth of surface degradation is inversely related to the gap voltage, at equal current set points, saturating around +500 mV. The most pronounced surface degradation is observed when lower bias voltages are applied to the tip. Considering that the surface features possess an electronic gap of roughly 700 mV, a small tip bias results in very close tip–sample distances, which can potentially influence the crystal surface’s covalent bonds. Nevertheless, after degradation occurs and the subsurface is exposed, the gap diminishes somewhat, Figure 2e,f – and therefore an increasing LDOS and increasing tip–sample distance - but the degradation continues. We can therefore exclude the tip physically milling away surface material as the dominant origin of the surface degradation under bias voltage variation at equal current set points. This persistence of degradation is evidenced by repeatedly scanning the same area, as shown in Supplementary S3, indicating that the degradation depth nearly doubles after a second scan.

To further assess the impact of electric field-induced degradation, we used non contact (nc)-AFM. In Supplementary S4, the AFM tip moves in proximity to the surface, comparable to the distance between the STM tip and the sample. The electric field is applied to the tip without permitting any tunnel current to flow between it and the sample. We observed a decrease in surface degradation; multiple scans were necessary to remove unit cell heights from the surface. Although the AFM scanning conditions resemble those of STM, the degradation strength is lessened, similar to the damage observed outside the STM scanning area in Figure 2a,g. These findings imply that the tunnel current plays the main role in the degradation strength; however, it is triggered by the polarity of the bias voltage and the gradient of the electric field between the tip and sample.

**Figure 4:**
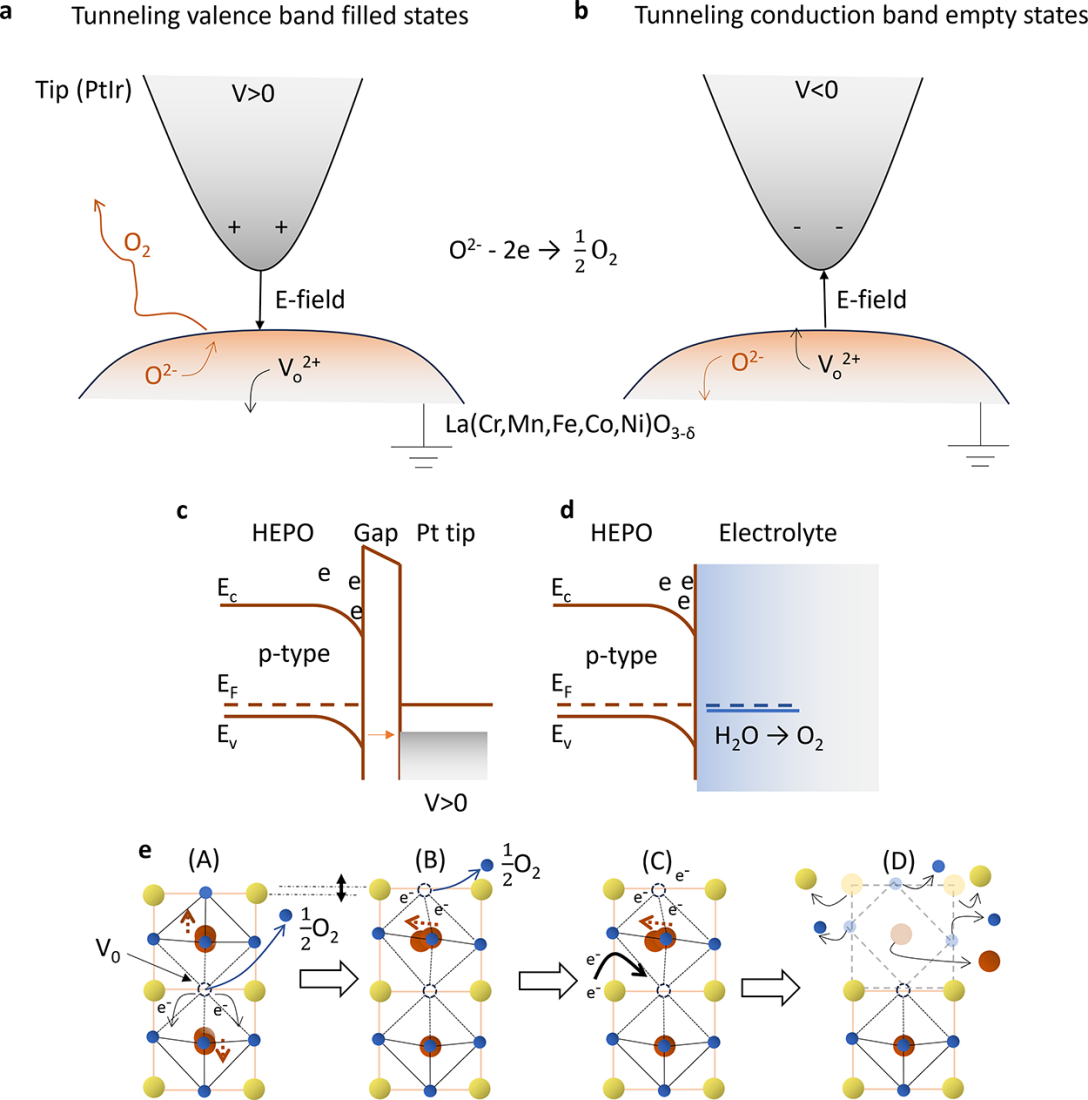
Illustration of the surface degradation process by STM and OER. (a, b) Illustration of the tip bias leading to oxygen extraction from the lattice and mobility of oxygen vacancies in the HEPO surface. The polarity of the bias *V* enables oxygen extraction for positive values. Tunneling into the filled states or empty states is indicated. (c, d) Schematic model of the p-type character of the HEPO film surface and energy band bending under positive voltage of the STM tip. In OER, similar downward energy band bending, at equilibrium of the Fermi level *E*_*F*_, leads to additional energy barrier for hole transport in the valence band *E*_*v*_ and accumulation of electrons in the conduction band *E*_*c*_, near the catalyst-electrolyte interface. (e) Schematic illustration of the degradation process. (A) The HEPO lattice experiences strain due to the five element B-site cations, leading to easy oxygen vacancy formation and diffusion. (B) Further distortion of the unit cell leads to increased formation of vacancies. Such distortion may be induced by the STM tip bias field leading to the oxygen anion extraction. (C, D) Charge trapping near the defects or vacancies leads to eventual collapse of the unit cell due to excessive charge-induced anisotropic strain. The elements are able to form new phases on the surface during the OER, such as La(OH)_*x*_.

We can project the current-dependency of the STM-induced degradation experiments to OER conditions, where degradation of the catalyst is influenced by the current magnitude of the reaction. The HEPO surface exhibits a noticeable p-type behavior as measured by STM STS. This contrasts with the bulk, which has been previously identified as an insulator at room temperature.^2^ When a positive bias voltage is applied to the STM tip, it results in downward bending of the HEPO surface energy levels, as depicted in Figure 4c. This causes electrons to be trapped in the conduction band gap interface, resulting in degradation as discussed subsequently. The downward band bending obstructs efficient hole and electron transfer, which leads to similar electron accumulation, as elaborated by Kibria et al.^41^

We now turn to the mechanism of tip-induced degradation. Oxygen vacancies, as for example discussed by Tyunina et al.,^42^ occur in epitaxially strained metal oxide perovskites which can induce a significant anisotropic lattice strain within the lattice surrounding the vacancy. The B-site cation can experience modified bond strength interactions with surrounding oxygen leading to local stress in the lattice, as schematically illustrated in Figure 4e-A. This lattice distortion probably controls not only the bond stability but also the electron trapping rate at the vacancy defect, leading to further structural instability by localized charges in the lattice and extraction of molecular oxygen by an electric field Figure 4e-B. Excess charge released by the formation of the oxygen vacancy can introduce structural deformations on its own, especially if the material is highly polarizable,^26^Figure 4e-C. The downward bending of the energy band on a p-type HEPO surface under the STM tip at positive bias voltage, promotes the trapping of charge, which likely cause structural instability and eventual collapse of the lattice, as the electron density rises,^43^Figure 4e-D.

The significant alteration in LDOS due to oxygen vacancy manipulation in HEPO thin film could be advantageous for device applications.^44^ Using an STM tip is a valuable tool to nucleate and probe the formation of oxygen vacancies on the surface of HEPO, and other metal oxide perovskite,^31^ films, which is rather difficult to probe with XPS only.^45^

### Post-OER Characterization

#### XPS Valence and SPM Characterization

The tip-induced degradation can be interpreted as an indication of tendency for activity evolution by surface transformation during OER of the HEPO thin-film catalyst. Consequently, we conducted a study to characterize how the film’s properties change after OER. To initiate the (in)stability of the catalyst, we conducted 20 cycles of *I*(*V*) and *CV*, with the results presented in Supplementary S5 and additional experimental details found in Methods. The *I*(*V*) results indicate an initial consistent downward shift in activity, or increase in overpotential η, with each subsequent cycle, beginning at η ≈ 400 mV, at a current density of 0.5 mA cm^–2^. Eventually, the catalyst stabilizes in activity after 15 cycles, albeit at a heightened overpotential of η ≈ 470 mV. Evaluating the catalyst solely through *I*(*V*) cycles poses challenges, as for example gas bubbles can deter activity and mask the true intrinsic performance.^46^ Thus, we analyzed the catalyst’s properties after the OER mainly by XPS and compared them with those of the initial state before the OER. Naturally, this introduces challenges in directly evaluating the potential changes in the catalyst’s properties that may occur during the OER. Nonetheless, significant differences in structure and chemical composition are observed to persist even after removing the catalyst from the solution.

**Figure 5:**
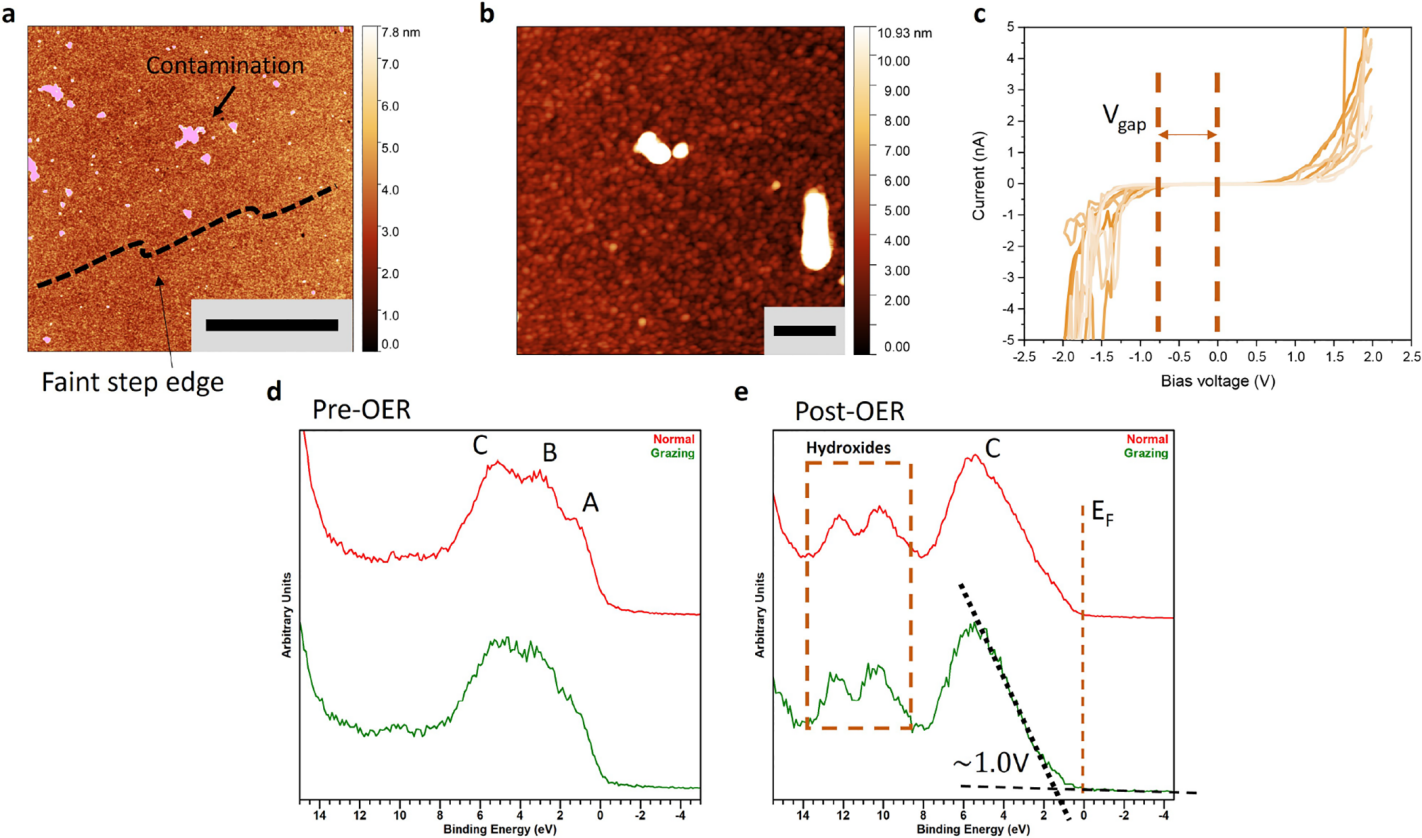
HEPO characterization post-OER (a) Large scale AFM image showing a rather featureless surface, with only a faint indication of step-edges of vicinal cut NSTO as indicated with the black dashed line. The purple colored features are likely of contamination origin. The scale bar is equal to 5 μm. (b) High-resolution AFM showing granular surface characteristics. The scale bar is equal to 200 nm. (c) STM *I*(*V*) curves showing the formation of a large surface electronic gap (*V*_gap_) of 0.8 V. (d, e) Pre- and post-OER XPS valence band spectra. The 3d-metal peak *e*_*g*_ and 2_tg_ (A, B) and the oxygen O_2p_ peak (C) are clearly distinguished. For post-OER additional peaks are formed at higher binding energies indicative of hydroxides indicated with the orange dashed box.

This activity reduction corresponds to a change in the surface morphology to the pre-OER catalyst, as investigated using AFM and STM, and given in Figure 5a,b. Prior, distinct step edges and a relatively flat surface morphology were noted. After, a more granular surface morphology became prevalent, resulting in a rather indistinct appearance, as shown in Figure 5a,b. The step edges became less defined and replaced by a uniformly distributed granular structure. The RMS roughness increased to a range of 360–460 pm. Locally, contamination-like characteristics, probably the result of OER deposition, were observed, as marked in purple in Figure 5a, and were not included in the RMS roughness calculation.

We performed *I*(*V*) tunneling spectroscopy on the surface following the OER. Figure 5d illustrates several *I*(*V*) spectra from different surface locations, demonstrating a significant electronic gap (*V*_gap_) near 0.8 V. This observed gap is larger compared to the original, pre-OER surface presented in Figure 2b and exceeds the gap measured in the degraded subsurface shown in Figure 2c. The surface analysis indicates the formation of a new phase, possibly an amorphous one, as previously reported for a comparable HEPO catalyst in ref (5).

XPS valence band spectra were analyzed to verify the formation of a new surface phase, as illustrated in Figure 5d,e. A marked decrease in the states near the Fermi level *E*_*f*_ was observed for the post-OER surface and a significant gap of approximately 1.0 eV, determined from the valence band of the grazing angle XPS, as denoted by the black dotted line in Figure 5e. This gap is consistent with the *V*_gap_ measured in Figure 5c. Moreover, a considerable change in the electronic structure was observed; of the peaks corresponding to *e*_*g*_ (peak A), t2g (peak B), and oxygen 2p (peak C) were visible in Figure 5e. For post-OER, as shown in Figure 5f, peaks A and B disappeared, signifying the breakdown of the 3d metal-oxide perovskite structure. The presence of some states near *E*_*f*_ in Figure 5e is attributed to a band tail.^47^ This band tail can arise from disorder induced by impurities, defects, and complete amorphization, leading to localized states within the band gap. Such band tail states may play a role in enhancing the effectiveness of lanthanum-based photocatalysts.^48^ The peak at 9.6 eV indicated within the dashed box in Figure 5e corresponds to La(OH)_3_,^49^ which did not occur in the pre-OER phase, Figure 5d. The left peak at 12 eV is likely also a metal (oxy)hydroxide species formed, like Ni, Mn and/or Co (oxy)hydroxide.

#### Post-OER XPS of the 3d-Metals and Oxygen Core Levels

**Figure 6:**
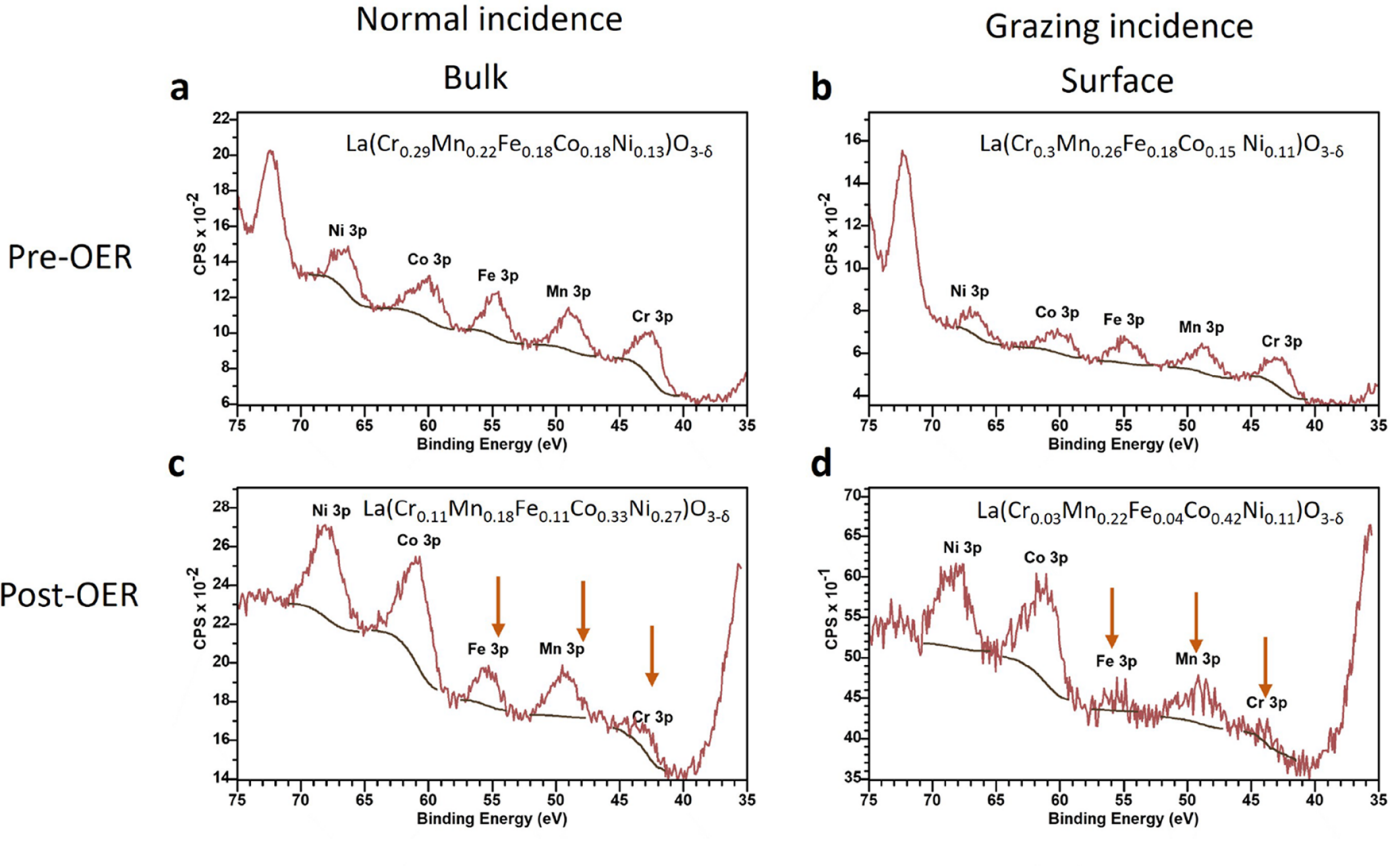
XPS spectra of the 3p transitions of the B-site metals pre- and post-OER (a, b) XPS spectra of the 3p orbital transitions for the B-site metals indicating minimal variation in chemical composition between bulk sensitivity (normal incidence) and surface sensitivity (grazing incidence) pre-OER. (c, d) Post-OER, a significant change in chemical composition of the B-site metals is measured. Fe, Mn and Cr have reduced stoichiometry (orange arrows) while an excess of Ni and Co enriches the surface.

XPS measurements reveal a notably alteration in the chemical composition of the film post-OER cycling. In Figure 6, the XPS-extracted stoichiometry of the 3d elements is given. Prior to OER, there is a small enrichment of Cr and Mn and some deficiency of Co and Ni. After the OER reaction the chemical composition of the 3d transition metal is altered. In Figure 6 it can be seen that the 3p peaks have shifted for most of the 3d elements the binding energy (BE) is increased except for Cr. The pre-OER BE values are in line with the air-exposed values in the work of Kante et al.^9^ for a comparable HEPO system. The shift to higher BE’s, which simply may interpreted as a larger valency, although for the complex strongly electron correlated, hybridized TM oxides^50,51^ the role of the O-2p states is comparably important. One may state, while oxygen vacancies rapidly alter the electronic configuration within the 3d TM element and O-orbital manifolds, the covalency changes drastically. The higher BE position of the core-level-3p state, as observed, is consistent with the formation of oxygen vacancies, giving rise to a localization of the TM valence 3d-electrons, hence a higher 3p-state BE value. However, there is no significant change in the XAS spectra for the Co-L edge for the operando OER experiments on similar films by Mueller et al.,^51^ so most probably an evolution of the surface composition took place. The surface shows a strong enhancement of Ni and Co in Figure 6, which may be do to enrichment at the surface driven by oxygen vacancy formation. A downward shift of the Co-L XAS lines after “near-OER” conditions^16^ indicates an increased 3d electron contribution at the Co site or in combination with the formation of metal-(oxy)hydroxides on the surface.^16^

These newly formed surface species can demonstrate even higher catalytic activity compared to the precatalyst, such for Ni-(oxy)hydroxides.^52^ In contrast to La(OH)_3_ formation on the surface, which is less catalytically active.^53^ Fe and Cr are less likely to generate such active surface species as they could dissolve in the electrolyte in highly alkaline environments.^54,55^ Thus, the B-site 3d metals Ni, Co, and Mn are interpreted to form (oxy)hydroxides that remain stable at the surface/electrolyte interface. The relative increase of Co can be attributed to diffusion from the bulk to the surface, forming also a metal-(oxy)hydroxide. The notable increase in the XPS signal counts of the Ni-, Co-3p state following the OER is intriguing, Figure 6d. The surface has become a particle-like structure visible in high densities in the post-OER AFM image, Figure 5b, which suggests atom compaction compared to the pre-OER perovskite structure, which is potentially the origin of the enhanced intensity of the XPS signal of the two 3d metals, Figure 6d.^56^

**Figure 7:**
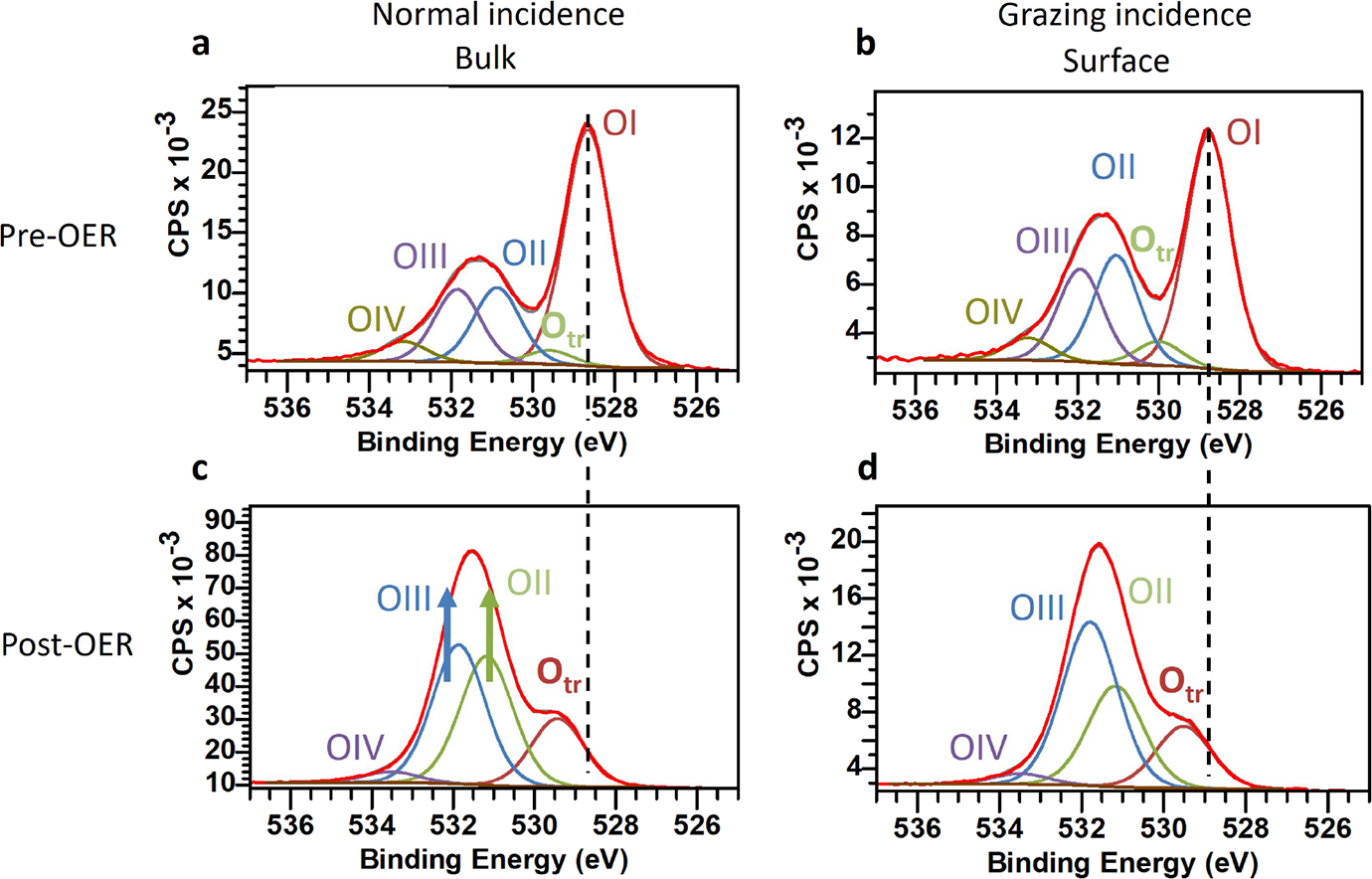
XPS spectra evolution of the oxygen 1s core level. (a, b) Pre-OER O 1s measured at normal incidence and grazing incidence, respectively. The lattice oxygen (OI) is assigned to the perovskite oxygen structure. The hydroxides measured are split in two, which can be assigned to mixed hydroxides (OII, OIII). A small amount of water is noted (OIV). (c, d) The post-OER spectra show considerable reduction in perovskite lattice oxygen (OI). The perovskite lattice has collapsed and oxygen is bound in metal(oxy)hydroxides (O_tr_). A strong increase of mixed hydroxides (OII and OIII) is noted, consistent with the hydroxination of the surface. The collapse of OI between pre-OER and post-OER is indicated by the dashed vertical line.

The participation of lattice oxygen is believed to significantly influence OER catalysis^57^ and be involved in surface transformations. Hence, the XPS O 1s spectra are measured to detect the various metal–oxygen transformations,^49^ and the results given in Figure 7. Because of ambient exposure prior to STM, some (oxy)hydroxides and water were measured by the XPS prior-OER, Figure 7a,b, indicated by OII, OIII and OIV with the oxyhydroxides lattice oxygen (O_tr_) at 529.5 eV and the lattice oxygen OI at 528.3 eV. We conducted XPS at both normal and grazing incidence to examine surface changes distinct from the bulk. Upon comparing pre- and post-OER in Figure 7a,c, it is evident that the lattice oxygen peak (OI) is strongly diminished compared to O_tr_, further support for significant chemical alteration is also the notable decrease in lattice oxygen (OI) concentration relative to other mixed hydroxide species (OII–OIII) as seen in Figure 7c,d. Complemented with the modifications of valence band spectra in Figure 5d,e, this points as well to a collapse of the 3d metal-oxide structure and an increased hydroxide contribution. This decrease in lattice oxygen is especially prevalent on the surface, Figure 7d, as expected for a surface transformation and is consistent with a oxygen to hydroxide transformation of the lattice. Peaks associated with the 3d-metal mixed hydroxide and La(OH)_3_ species (OII, OIII) have indeed increased. Due to ambient exposure, more physisorbed hydrocarbons with hydroxide groups (OIII) are present on the surface, Figure 7d.

**Figure 8:**
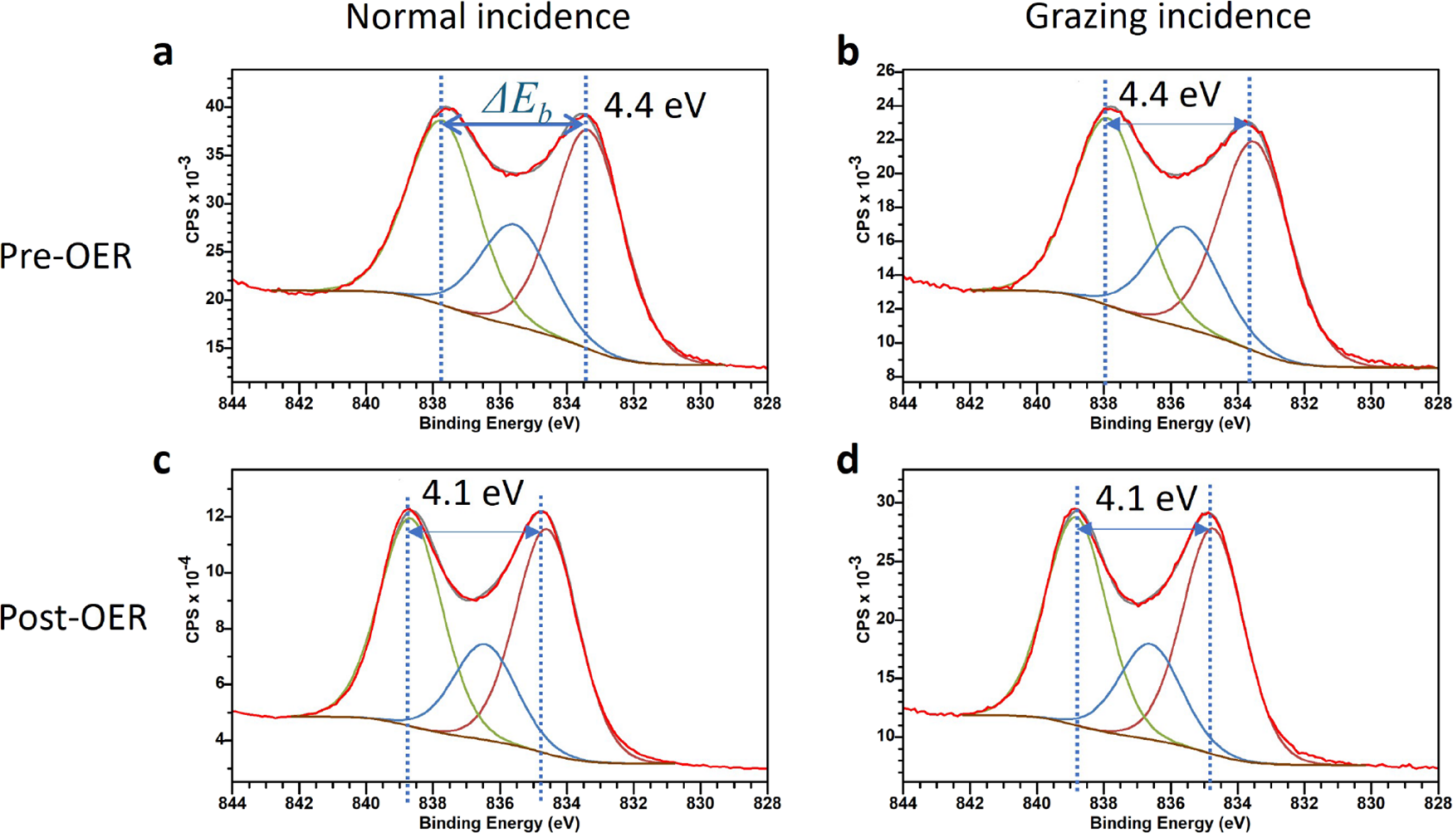
XPS spectra of the La 3d5/2 transitions. (a, b) XPS spectra of the La 3d5/2 transition pre-OER. (c, d) Post-OER spectra taken at normal and grazing incidence. The shift and magnitude change of Δ*E*_*b*_ is given indicating a shift from La_2_O_3_ character to La(OH)_3_ as a surface transformation.

To confirm the transformation of La_2_O_3_ to La(OH)_3_ after the OER, the difference in binding energy (Δ*E*_*b*_) between the two peaks, mainline and satellite, of the La 3d5/2 spectra in Figure 8 corresponds to the Δ*E*_*b*_ of 4.6 eV for La_2_O_3_ and 3.9 eV for La(OH)_3_ respectively, reported by Sunding et al.^49^ The slight difference in splitting of 0.2 eV of the 3d5/2 peak compared to La_2_O_3_ and La(OH)_3_^49^ is likely related to differences in the degree of crystallinity and strain effects compared to the literature values for crystalline materials, as the satellite position is very sensitive to the 3d5/2cf̲^1^-*O̲*2p bonding configuration.^49^ Comparable values, 4.1 and 3.8 eV for pre- and post-OER, respectively, have been discussed by Weber et al.^16^ for La_0.6_Sr_0.4_CoO_3−δ_. The upshift of ∼ 1 eV of the La-3d5/2 for the post-OER spectrum in Figure 8 compared to the pre-OER spectrum resembles the almost complete loss of the lattice O-1s peak at 528.6 eV in the pre-OER spectrum in Figure 7a, providing only the O-1s contribution of the oxyhydroxides lattice at 529.5, ∼1 eV higher, available for the bonding with La-3d electrons.

## Conclusions

We observe significant surface transformations of high entropy perovskite oxide La(Cr,Mn,Fe,Co,Ni)O_3–*δ*_ epitaxial OER catalysts utilizing a complementary approach of SPM and XPS. During OER, the surface undergoes structural reorganization, resulting in a layer enriched with La(oxy)hydroxide. The surface concentrations of other 3d elements such as Fe, Mn, and Cr are notably reduced, likely due to diffusion during OER. We attribute the cause of the chemical surface changes to the leaching of oxygen anions and accumulation of vacancies on the surface, with the latter acting as charge-trapping sites. These sites and the trapped charges influence bond strengths within the crystal and likely contribute to the lattice stability, eventually leading to the dissolution of elements into the electrolyte and restructuring of the surface. We have shown that STM is a viable technique for studying possible origins of surface transformations of epitaxial catalysts even at pre-OER conditions. For future studies, without a clear post-OER analysis of HEPO catalysts that rules out surface transformations, potential beneficial surface transformations should be further explored, for example by tuning the molar ratio of the 3d-metals. The process of chemical dissolution, such as with Sr doping, has the potential to increase the surface reconstruction rate, resulting in a highly active high-entropy catalyst.^58^ However, the electrocatalyst and its surface transformations studied here may not be applicable to all high-entropy oxide catalysts. It is crucial to note that this study focused on an epitaxial thin film as a model system, which may suffer from lateral resistivity limitations.^59^ Catalysts composed of nanoparticles might offer enhanced stability, using chemical protection of Ni cores using chromium oxide, for example.^60^ Theoretical understanding of the link between local lattice distortion and vacancy formation is promising.^61^ Furthermore, in operando studies using electrochemical STM^29^ and XPS are valuable for uncovering the dynamics of surface transformations.

## Methods

### Sample Fabrication

The sample fabrication is given in ref (9).

### Electrochemical Characterization

We performed electrochemical tests on epitaxial thin films on 10 × 10 × 0.5 mm^3^ Nb:SrTiO_3_ single crystal substrates, embedded in a custom sample holder. This configuration enabled secure attachment of the sample’s rear to the Pt plug of a rotating disk electrode (RDE, Pine Research). Additionally, 50 nm thick Pt connections facilitated the electrical linkage from the back to the front, connecting the Nb:SrTiO_3_ substrate with the epitaxial film. On the front, a film area with a 7.5 mm diameter was exposed to the electrolyte, sealed effectively with an O-ring (Kalrez, ERIKS, Germany). The RDE shaft was set to rotate at 1600 rpm. A Biologic SP-200 potentiostat was used for electrochemical measurements, involving cyclic voltammetry at a sweep rate of 10 mV s^–1^, within a 150 mL Teflon cell resistant to alkaline substances (Pine Research). This setup incorporated a Pt wire as the counter electrode. Electrochemical impedance spectroscopy (EIS) was performed with an amplitude of 20 mV at the open circuit potential, including IR correction (typically between 45–55 Ω) derived from the high frequency intercept of the real impedance. The electrolyte, a 0.1 M KOH solution, was prepared by dissolving high-purity KOH pellets (Sigma-Aldrich, 99.99%) in Milli-Q deionized water (resistance >18.2 M Ωcm). It was saturated with O_2_ for a minimum of 30 min before experimentation and kept in an oxygen atmosphere during tests. All electrochemical measurements were taken at room temperature to ensure consistency and comparison with recommended model electrocatalyst system protocols.^62^ The potentials were measured against an Hg/HgO reference electrode (C3 Prozess- and Analysentechnik, Germany). Initial cyclic voltammetry was performed within the pseudocapacitive redox phase change window, approximately 0.9 to 1.75 V vs RHE, at scan rates from 10 to 500 mV s^–1^, followed by oxygen evolution reaction (OER) tests from 0.9 to 1.9 V vs Hg/HgO, conducted at a scan rate of 10 mV s^–1^. Capacitive effects in the CV data were adjusted by averaging the forward and backward scan results.

### Ambient AFM

Tapping-mode AFM was performed using a Nanosensor NCH-PPP Si cantilever on a Veeco Dimension III microscope under ambient conditions. Imaging was carried out with a scan speed of 1 lines per second (512 lines/512 pixels). Postprocessing of the AFM data was performed with Gwyddion software.^63^ Images were line aligned using median of differences and plane leveled using mean subtraction.

### Non Contact AFM

Non contact AFM was performed with a Scienta Omicron GmbH VT-SPM operating in an ultra high vacuum of 10^–10^ mbar at room temperature. A qPlus sensor (Scienta Omicron GmbH) with an etched tungsten tip was used. The bias was applied to the tip and the sample grounded. The cantilever oscillated at a frequency of 25 kHz. The postprocessing of the data was performed with Gwyddion software.^63^ The images were line-aligned using median of differences and plane-leveled using mean subtraction.

#### UHV STM and STS

Scanning tunneling microscopy was performed with a Scienta Omicron GmbH VT-SPM operating in an ultra high vacuum of 10^–10^ mbar at room temperature. STM tips were mechanically cut from PtIr wire. The bias was applied to the tip, and the sample was grounded. The imaging was performed in constant current mode. For d*I*/d*V* mapping and spectroscopy, an Stanford Research Systems lock-in amplifier SR844 with an alternating voltage between 20 mV and 100 mV applied at 3.5 kHz. The postprocessing of the STM data was performed with Gwyddion software.^63^ The images were line-aligned using median of differences and plane-leveled using mean subtraction.

### XPS

XPS spectra were acquired with a K-Alpha XPS system (Thermo Scientific) and an ESCALab II system. The first instrument utilizes Al kα radiation with an energy of 1486.6 eV, with a monochromator employed. The second instrument uses Mg Kα radiation with an energy of 1253 eV. To obtain an adequate signal-to-noise ratio, 30 scans were collected and averaged for the O 1s spectra, while 50 scans were taken for the metal and valence spectra. The dwell time per measurement point was set at 50 ms. CasaXPS software was used for spectral analysis. Shirley background subtraction was employed for selected regions, and peak fitting was conducted with GL(30) line shapes, which represent a composition of 70% Gaussian and 30% Lorentzian line shapes. The full width at half-maximum (FWHM) was consistent among similar species for deconvolution purposes. For quantitative evaluation, relative sensitivity factors (RSF) specific to this instrument were utilized. RSFs provided a means to compare transitions across various orbitals and elements by adjusting for the photoionization cross-section and the electron mean free path.

Next to normal incidence, grazing incidence XPS was measured with an angle of 30° between sample and detector, compared to an angle of 90° between sample and detector for normal incidence. The samples were mounted on the sample holder using double sided carbon tape for the grazing incidence XPS measured, for the normal incidence measurement the samples were clamped with brackets and did not need further attachment using carbon tape to the sample holder.

The spectra are adventitious carbon corrected. This correction has an associated uncertainty of ± 0.2 eV. Quantitative analysis of the 3p B-site metals XPS spectra is used to verify the stoichiometry.

### XRD

XRD measurements were carried out in a Bruker D8 Advance system using monochromated Cu Kα_1_ and a 0.2 mm slit on the primary beam path. The scattered X-rays were received under a 0.25 mm slit and measured by an EIGER2 R 250 K detector in 0D mode.

## Supporting Information Available

Tip radius dependent degradation, HEPO surface degradation as a function of tip bias voltage, HEPO surface degradation by STM bias value and repeated scanning, AFM degradation, and cyclic voltammetry dependent activity change (PDF)
