# Supplementary material for: Oxygen Vacancies Can Drive Surface Transformation of High-Entropy Perovskite Oxide for the Oxygen Evolution Reaction as Probed with Scanning Probe Microscopy
Source: ACS Appl Mater Interfaces. 2025 Apr 3;17(15):23237–48. doi: 10.1021/acsami.4c22352 (PMC12012691; doi:10.1021/acsami.4c22352)
Supplement: Supplementary file 1 — am4c22352_si_001.pdf [file am4c22352_si_001.pdf]

Oxygen vacancies can drive surface transformation of high-entropy perovskite oxide for the oxygen evolution reaction as probed with scanning probe microscopy - Supporting Information

Michael Verhage, Stijn van den Broek, Christ Weijtens, and Cornelis F.J. Flipse*

Molecular Materials and Nanosystems (M2N) - Department of Applied Physics and Science Education - Eindhoven University of Technology, Eindhoven, Netherlands

E-mail: c.f.j.flipse@tue.nl

Supplementary S1

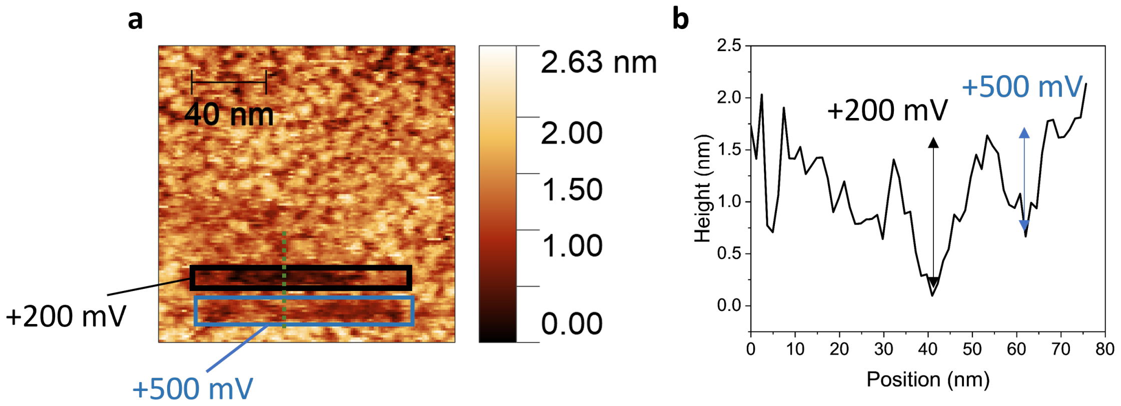

Figure S1: **HEPO degradation probed with increased STM imaging resolution.** (**a**) 150x150 nm image, captured at a -500 mV bias voltage, revealing two horizontal regions with surface damage caused by negative bias scanning. The tip's radius R was smaller than that in the main text, evidenced by the enhanced contrast imaging of the corrugation features on the surface. The area within the black box was scanned with a +200 mV bias, while the highlighted region within the blue box was scanned with a +500 mV bias. (**b**) A line trace along the dotted line in (a) displays two damaged regions. The sharper STM tip results in reduced damage extending beyond the scanned area at positive tip bias, which relates to a more localized electric field between the tip and sample surface.

Supplementary S2

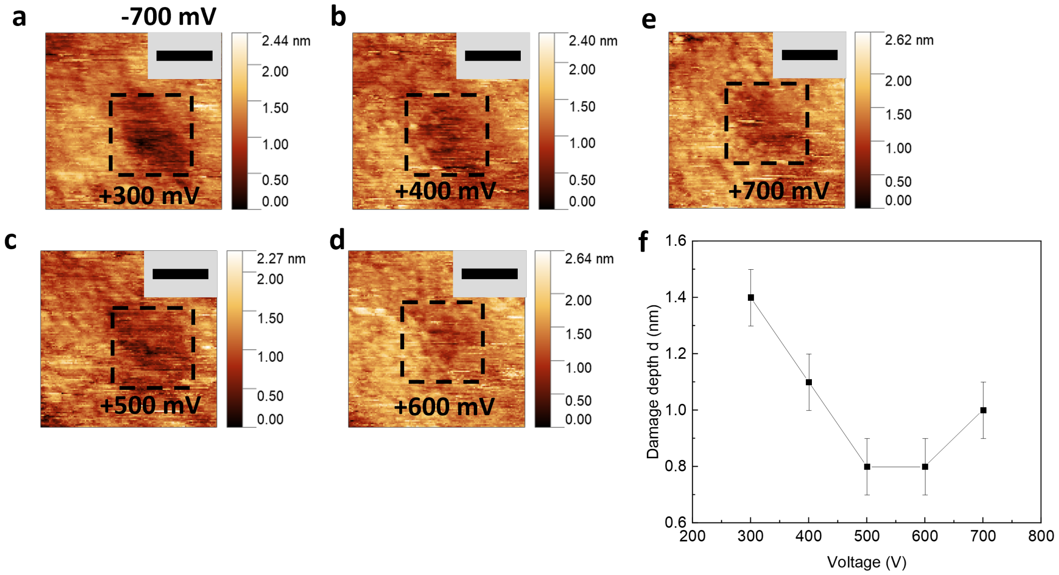

Figure S2: **HEPO surface degradation as function of tip bias voltage.** (**a-d**) Large scale, 100x100 nm, images taken at bias of +700 mV and a tunnel current of 300 pA. The black dashed line show area's scanned with an increasingly larger tip bias prior to the large scale images. The tip-induced degradation is visible in the black dashed boxes. (**f**) The depth of surface degradation scales inversely with the tip bias voltage strength and saturates at +500 mV. The black scale bar is equal to 30 nm.

Supplementary S3

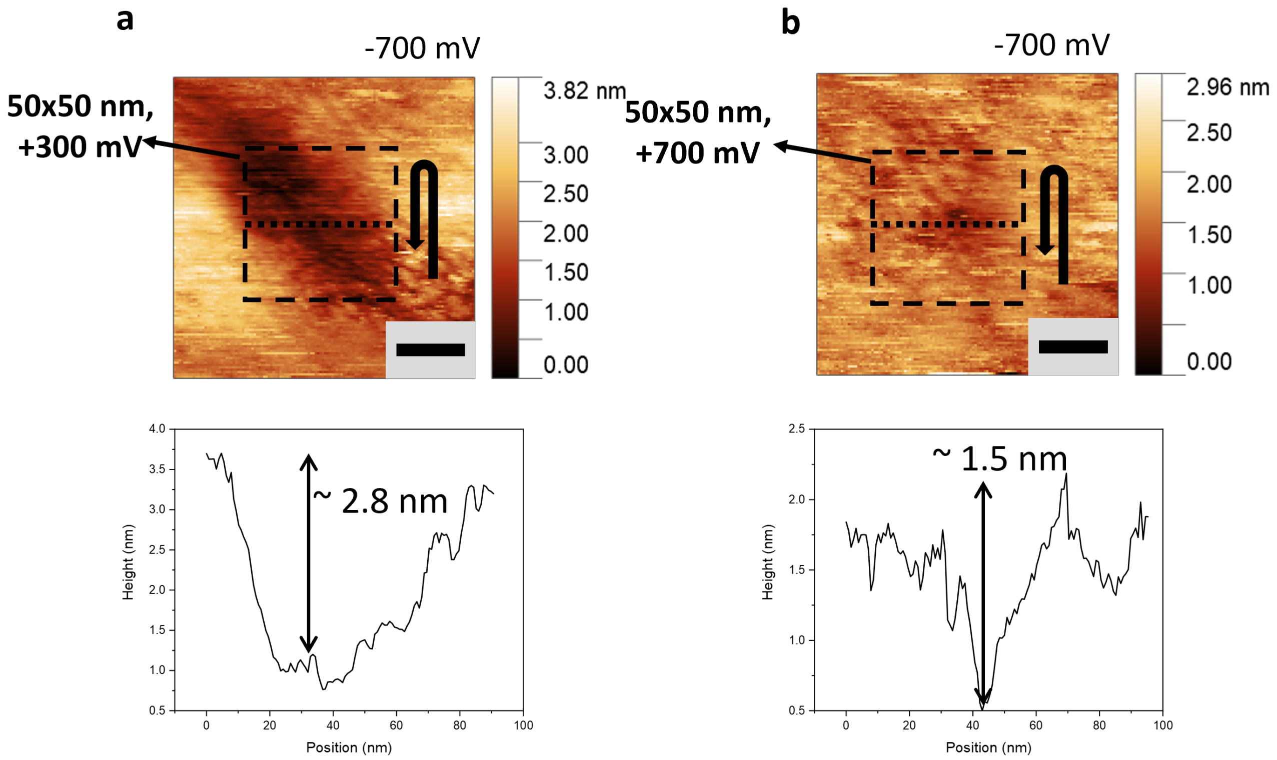

Figure S3: **HEPO surface degradation by STM bias value and repeated scanning.** (**a**) Large scale, 100x100 nm, imaged at bias of +700 mV and tunnel current of 300 pA. The image shows local degraded surface imaged with negative bias of +300 mV in a 50x50 nm area, scanned twice. The imaged was scanned twice to increase the total depth damage from 1.4 nm to 2.8 nm. A trace profile is indicated with the horizontal dotted line and given below the image. (**b**) Similar experiment to (a) but with an increase negative bias voltage of +700 mV in the 50x50 nm area. The total depth damage is reduced to 1.5 nm. Increased negative bias imaging reduced surface degradation strength. The black scale bar is equal to 20 nm.

Supplementary S4

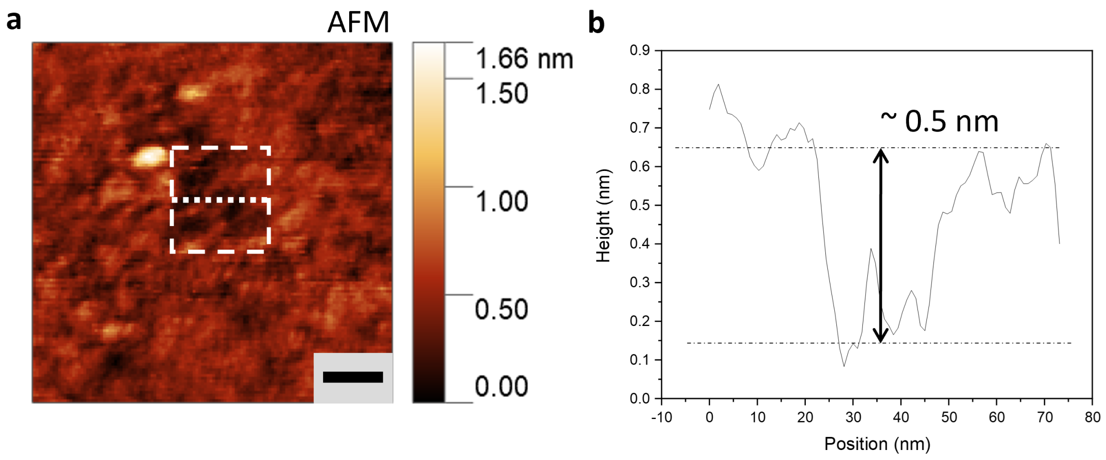

Figure S4: **AFM degradation** (**a**) nc-AFM image showing similar surface imaging as obtained by STM. Locally, damage to film can be induced within the withe dased square, without allowing a tunnel current to flow. A bias of +300 mV has been applied to the tip. The overal magnitude of damage is reduced compared to STM. About one unit cell of surface has been degraded by the tip, as can be seen in the height trace in (**b**), from the white dotted line in **a**. The black scale bar is equal to 50 nm.

Supplementary S5

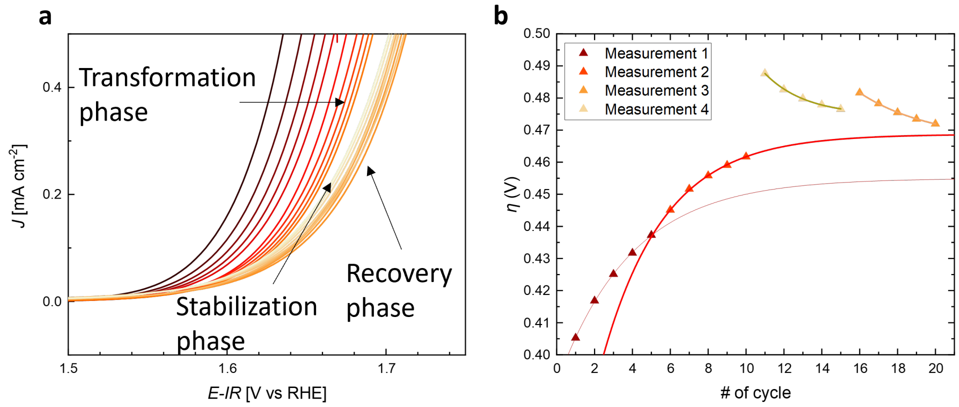

Figure S5: **Cycle-dependent change in activity.** (**a**) Throughout the initial 10 $I(V)$ cycles, the activity diminishes in a non-monotonic manner, coinciding with the occurrence of surface transformation. Eventually, the phase of decrease in activity or recovery results in a steady level of activity approximately at cycle 15. (**b**) Overpotential η evolution as function of cycle. The overpotential is derived from the standard OER potential of 1.23 V, combined with the potential relative to the RHE at a current density of $0.5\,\mathrm{mA\,cm^{-2}}$.
